# A community effectiveness trial of strategies promoting intermittent preventive treatment with sulphadoxine-pyrimethamine in pregnant women in rural Burkina Faso

**DOI:** 10.1186/1475-2875-7-180

**Published:** 2008-09-18

**Authors:** Sabine Gies, Sheick Oumar Coulibaly, Florence Tiemegna Ouattara, Clotilde Ky, Bernard John Brabin, Umberto D'Alessandro

**Affiliations:** 1 Epidemiology and Control of Parasitic Diseases Unit, Department of Parasitology, Institute of Tropical Medicine, Antwerp, Belgium; 2 UFR Sciences de la Santé, Université de Ouagadougou, Burkina Faso; 3 Laboratoire National de Santé Publique, Ouagadougou, Burkina Faso; 4 District Sanitaire Boromo, Burkina Faso; 5 Child and Reproductive Health Group, Liverpool School of Tropical Medicine, Liverpool, UK; 6 Emma Kinderziekenhuis, Academic Medical Centre, Amsterdam, Netherlands

## Abstract

**Background:**

Intermittent preventive treatment with sulphadoxine-pyrimethamine for pregnant women (IPTp-SP) is currently being scaled up in many countries in sub-Saharan Africa. Despite high antenatal clinic (ANC) attendance, coverage with the required two doses of SP remains low. The study investigated whether a targeted community-based promotion campaign to increase ANC attendance and SP uptake could effectively improve pregnancy outcomes in the community.

**Methods:**

Between 2004 and 2006 twelve health centres in Boromo Health District, Burkina Faso were involved in this study. Four were strategically assigned to community promotion in addition to IPTp-SP (Intervention A) and eight were randomly allocated to either IPTp-SP (Intervention B) or weekly chloroquine (Control). Primi- and secundigravidae were enrolled at village level and thick films and packed cell volume (PCV) taken at 32 weeks gestation and at delivery. Placental smears were prepared and newborns weighed. Primary outcomes were peripheral parasitaemia during pregnancy and at delivery, placental malaria, maternal anaemia, mean and low birth weight. Secondary outcomes were the proportion of women with ≥ 3 ANC visits and ≥ 2 doses of SP. Intervention groups were compared using logistic and linear regression with linearized variance estimations to correct for the cluster-randomized design.

**Results:**

SP uptake (≥ 2 doses) was higher with (Intervention A: 70%) than without promotion (Intervention B: 49%) (OR 2.45 95%CI 1.25–4.82 p = 0.014). Peripheral (33.3%) and placental (30.3%) parasite rates were significantly higher in the control arm compared to Intervention B (peripheral: 20.1% OR 0.50 95%CI 0.37–0.69 p = 0.001; placental: 20.5% OR 0.59 95%CI 0.44–0.78 p = 0.002) but did not differ between Intervention A (17.4%; 18.1%) and Intervention B (20.1; 20.5%) (peripheral: OR 0.84 95%CI 0.60–1.18 p = 0.280; placental: OR 0.86 95%CI 0.58–1.29 p = 0.430). Mean PCV and birth weight and prevalence of anaemia and low birth weight did not differ between study arms.

**Conclusion:**

The promotional campaign resulted in a major increase in IPTp-coverage, with two thirds of women at delivery having received ≥ 2 SP. Despite lower prevalence of malaria infection this did not translate into a significant difference in maternal anaemia or birth weight. This data provides evidence that, as with immunization programmes, extremely high coverage is essential for effectiveness. This critical threshold of coverage needs to be defined, possibly on a regional basis.

## Background

Each year, about 50 million women living in malaria endemic regions become pregnant, more than half in sub-Saharan Africa [1]. In areas of relatively stable transmission, where acquired immunity to *Plasmodium falciparum *limits infection and prevents severe disease in adults, women in their first and second pregnancy are the most vulnerable [2], due to a higher risk of severe anaemia and a low birth weight (LBW) outcome, a leading cause of child mortality and poor growth and development [3-7].

Malaria in pregnancy and its adverse consequences can be prevented with suppressive antimalarial treatment or chemoprophylaxis [8]. Weekly chloroquine (CQ) has been the basis for prevention for many years, but now has limited application. There are difficulties in coverage and compliance throughout pregnancy and parasite resistance to CQ has effectively made its use redundant in *P. falciparum *endemic areas [9,10]. A new strategy for prevention based on insecticide-treated bed nets (ITNs) and use of intermittent preventive treatment in pregnancy (IPTp) has been formulated [1,11]. IPTp is based on the administration of treatment doses of sulphadoxine-pyrimethamine (1500/75 mg; SP) to all pregnant women at pre-defined intervals and regardless of malaria infection. Currently, WHO recommends administering SP two or three times at scheduled antenatal visits at least one month apart from the second trimester onwards [1]. Evidence of the efficacy of IPTp with SP for preventing malaria infection and improving birth weight was reported from East Africa [3,12,13] and West Africa [14,15].

IPTp assumes that the large majority of pregnant women attend antenatal clinics (ANC) at least twice during their pregnancy and at a time when SP can be administered under direct observation. Unfortunately, late attendance to ANC and weak health services limit the effectiveness of this strategy [16-19]; coverage with two or more SP doses varies widely (24–68%) [20] and is well behind the goal of 80% proposed by the Roll Back Malaria Partnership [11,21]. New approaches to increase IPTp coverage are urgently needed.

In Burkina Faso, CQ and SP have been used as first and second line treatment for uncomplicated malaria until 2005. Parasitological resistance to CQ was 18% in children in Bobo-Dioulasso in 1998–2000 [22] while adequate clinical and parasitological response (ACPR) in pregnant women in Ouagadougou was 53.3% (Day 28) in 2003 [23]. Resistance to SP was similar in children and pregnant women in 2003 with ACPR (D28) of 91.8% and 87.1% respectively [23,24].

This study investigated whether promoting regular and early antenatal attendance of pregnant women through community based health education would increase coverage and uptake of IPTp and consequently improve pregnancy outcomes. In addition, the effectiveness of IPTp-SP compared with weekly CQ was determined in order to provide additional evidence to the Burkinabe Ministry of Health for an impending policy change.

## Methods

### Study site

**Figure 1:**
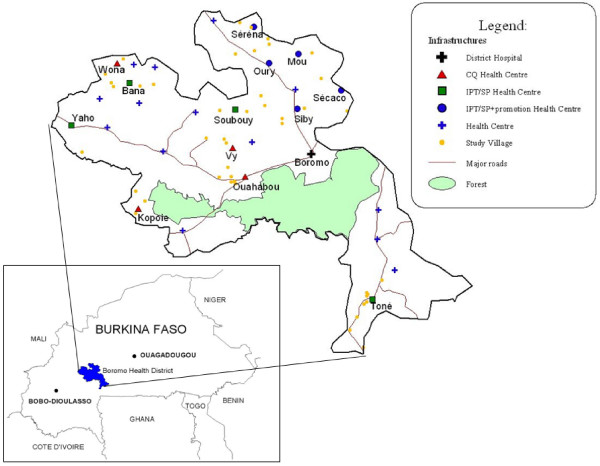
Location of study health centres and dependant villages in Boromo Health District, Burkina Faso.

The study was carried out between 2003 and 2006 in Western Burkina Faso, in Boromo Health District (BHD), a rural province with an estimated total population of 204,117 (Figure 1). There are three seasons: a rainy season (June to October; 20–35°C; mean annual rainfall about 800 mm/year), a cold dry season (November to February, 16–32°C) and a hot dry season (March to May, 25–40°C). Malaria is holo-endemic, with high transmission between July and December. At the time of the study, national guidelines for malaria prevention in pregnant women recommended a full treatment course of CQ (1500 mg over 3 days) at the first antenatal visit followed by 300 mg weekly until 6 weeks post partum. Antenatal care was offered free of charge and included, besides CQ prophylaxis, an ANC card, physical examination, counselling, and haematinic supplementation (200 mg ferrous sulphate and 0.25 mg folic acid). In rural Burkina Faso, antenatal coverage for at least one visit was about 70%, with 22.5% of first visits during the first trimester and 68.5% of deliveries occurring at home [25].

### Study design and randomization

Study interventions were implemented at two different levels: IPTp with SP (two observed doses at the beginning of the second and third trimester) was introduced through antenatal clinics in selected health centres (HC) and promotional activities were conducted at village level.

Four out of 26 peripheral HC in BHD were strategically assigned to community promotion in addition to IPTp-SP (Intervention A). Geographically contiguous HC were selected to avoid contamination due to the spread of the promotional campaign across the study arms (Figure 1). Communities were informed about the dangers of malaria for the pregnant women and their babies and early and regular ANC attendance was promoted to ensure timely IPTp-SP uptake. In 18 villages, female community leaders were trained to promote specifically designed health messages using image boxes for individual and group discussions. These messages were based on a previous socio-anthropological survey investigating local perceptions and beliefs.

Eight HC were randomly allocated to either implement IPTp-SP in antenatal clinics without these enhanced promotional activities (Intervention B) or continue with weekly CQ according to the national guidelines (Control).

The total study area covered a population of about 75,000 people distributed in 57 villages. Catchment areas of the selected HC varied in number of villages (2–10) and population size (3,500–10,500). In one catchment area (Oury), a new HC (Mou) was opened during the study period, reducing the distance to the nearest HC for two villages. SP was available at ANC from April 2004. The promotional activities in the intervention villages started in May 2004 and continued until June 2006. In August 2004, as part of an additional nutritional study, HC were in a factorial design assigned to one of two forms of micronutrient supplementation: (a) standard haematinics or (b) daily multi-micronutrients [26].

### Enrolment and follow-up

Trained women field assistants (WFA) identified pregnant women by monthly village visits using a screening questionnaire. After obtaining an informed consent, women in their first or second pregnancy were recruited. A questionnaire on demographic and household characteristics, education and socio-economic status, obstetrical history, antenatal visits, illness and treatment during the current pregnancy was administered by the WFA. Uterine fundal height was measured to confirm pregnancy and to estimate the gestational age. If the uterus was non-palpable, a urine pregnancy test was performed. Enrolled women received a card with a unique study number to be shown any time they attended a HC. At around 32 weeks of gestation, WFA visited the enrolled women and administered a questionnaire on antenatal visits, morbidity and treatment received. A capillary blood sample (finger prick) for packed cell volume (PCV) and parasitaemia was collected.

#### ANC visits

At each ANC visit, information on previous illnesses and treatments was collected; fundal height and axillary temperature were measured. Numbers of tablets of directly observed SP treatment and other medicines (CQ, haematinics) handed to mothers were recorded on a study questionnaire. Similar information was collected at unscheduled visits.

#### Delivery

About half of deliveries were expected to take place at home assisted either by a traditional birth attendant (TBA) or a family member. Around the expected time of delivery, WFA weekly visited women likely to deliver at home. As soon as possible after delivery, babies were weighed using a hanging weighing scale (UNICEF Scale, infant, spring, 5 kg × 25 g) and length was measured to the nearest half centimetre using a transportable measuring board (SECA 210 Measure Mat II). A capillary blood sample for PCV and parasitaemia was collected from the mother. Whenever possible, WFA cut a small piece of tissue from the middle third of the maternal side of the placenta and prepared a smear after swabbing it on blotting paper. Similar samples and information were collected from women delivering at a HC or district hospital in the study area.

#### Child survival

Women recruited in the study and their offspring were visited by WFA about one year after delivery; if the child had died, the time of the event and its circumstances were recorded.

### Laboratory investigations

All laboratory tests were performed by three experienced technicians in the laboratory of Boromo District Hospital.

#### Malaria parasitaemia

Thick films and methanol fixed placental smears were stained with 10% Giemsa for 10 minutes. For peripheral blood, parasite density was determined by counting parasite asexual forms per 200 white blood cells (WBC). The parasite density per μl was estimated assuming 8,000 WBC/μl. A slide was considered negative if no parasite was found after counting 500 WBC. All slides were systematically read by two technicians and for discrepant results a third consensus reading was performed. Parasite density for placental smears was expressed as the percentage of parasitized red blood cells (RBC) over the total number of RBC after counting at least 1,000 RBC.

#### Haematology

Heparinized capillary tubes containing whole blood were centrifuged within 48 hours after collection and PCV read. To minimize losses during the transport two capillaries were collected from the same finger prick. If two results were available the mean value was computed.

### Definitions

#### Malaria infection

Asexual *P. falciparum *parasites of any density, in a thick film of peripheral blood (peripheral parasitaemia) or a placental smear (placental parasitaemia).

#### Anaemia

PCV < 33%; women were further divided according to the degree of anaemia, i.e. moderate to severe anaemia PCV < 30%; severe anaemia PCV < 24%. For the analysis of the haematological status at delivery, only blood samples collected at the day of delivery were considered.

#### Birth weight

Weight values obtained within 24 hours of delivery were analysed as such. Weights obtained between day 1–8 post-delivery were corrected for the physiological fall (D1 4%, D2 3%, D3 3%, D4 1%) and increase (D5 0%, D6 1%, D7 2%, D8 4%) in weight occurring during the first week after delivery. The correction factor was estimated by weighing 132 newborns with known birth weight every two days up to 8 days after delivery. The birth weight analysis includes only singleton live births. Low birth weight (LBW) is defined as a corrected birth weight < 2,500 grams.

#### Gestational age

Fundal height measurements, when compared with delivery dates, were shown to be inaccurate for estimating the gestational age. Thus, gestational age at first antenatal visit was estimated using the time between first ANC visit and delivery [27], assuming delivery occurred after 40 weeks gestation. Women were considered in the first trimester when their first antenatal visit was 25 to 40 weeks before delivery, in the second if between 13 to 24 weeks and in the third if between 0 and 12 weeks. Analyses including gestational age were restricted to normal deliveries of singleton live-births. Due to the uncertainty of gestational age, no attempt to define preterm delivery and to distinguish between miscarriages and stillbirths was done.

#### Season

Low transmission from January through June and high transmission from July through December, based on locally obtained rainfall and malaria prevalence, and taking into account the delay between onset of rains and onset of malaria transmission. All deliveries and blood samples were thus assigned to a period of either "low" or "high" transmission. In addition, for birth weight analysis the low transmission period was sub-divided into "early" (January to March) and "late" (April to June) low transmission.

#### Wealth-index

A relative index of socio-economic status based on the construction material of the house and roof and household assets (bicycle, motorbike, cart, car, television, radio, cell phone) was constructed using principal component analysis [28,29]. Classes were derived by dividing the index into quartiles graded as most poor, poor, less poor and least poor.

#### Distance

Mean distances between villages and the nearest HC are those used by BHD for the vaccination program and are consistent with our own GPS derived data.

### Data analysis

#### Sample size

Assuming a mean birth weight of 2,600 grams with no intervention and accounting for the cluster (village) effect with a small coefficient of variation between clusters in each group, the study was designed to detect, at the 5% level and with 80% power, a difference in mean birth weight of 200 grams between study arms. With 50 expected pregnancies per 1,000 inhabitants per year, one third first or second pregnancies, and allowing for 20% loss to follow-up, a total population of 70,000 was needed for including and following up 2,000 primi- and secundigravidae in two years.

#### Statistical analysis

Access 2003 was used for double data entry and validation and EpiInfo 2000 (version 3.2.2; Centers for Disease Control and Prevention, Atlanta) and STATA (Intercooled version 10; Stata Corp., College Station, TX) software packages were used for analysis. Initially villages were intended to be considered as the sampling unit. However, as HC were the units of randomization, it was decided to account for the cluster design using linearized variance estimations with HC as the primary sampling unit (svyset HC, vce (linearized) in STATA 10). Baseline characteristics were compared between study arms using a design-based Pearson χ^2^-test (svy linearized: tabulate in STATA 10). Linear regression models (svy linearized: regress) were used to compare means of birth weight and PCV and logistic regression models (svy linearized: logistic) to compare proportions of ANC and IPTp-SP coverage, parasitaemia, anaemia and LBW and to determine odds ratios with corresponding 95% confidence intervals (CI). The criterion for statistical significance was set at alpha = 0.05. Variables tested as possible confounders were age (dichotomized ≤ 19/> 19 years), parity, education, marital status, wealth index, bed net ownership, season, distance (dichotomized ≤ 5/> 5 km) and, for birth weight analyses, sex of the baby. Variables associated with outcomes at a significance level with p < 0.1 in univariate analysis were entered in multiple logistic regression models. Intervention arm was kept as a variable in all models.

#### Final sample

Women enrolled during the first months of the study were likely to have started antenatal clinics before the interventions were implemented and have already received CQ chemoprophylaxis instead of IPTp-SP. The intervention A arm would not have been exposed to promotional activities. Therefore, women in their 4^th ^or later month of pregnancy at the time the study started (delivery date prior to September 1^st ^2004) were excluded *a priori *from the analysis.

### Ethical clearance

The study was approved by the Burkina Faso Ministry of Health and the Ethical Committee at ITM, Antwerp. Local health authorities and community leaders were informed about the study objectives and procedures for data collection. All study participants gave informed consent after explanation of the procedures in the local language and were free to remove consent at any time of the study without influencing their access to health services. Women found to be parasitaemic or anaemic at 32 weeks or at delivery were offered antimalarial treatment (either quinine in the intervention arms or CQ in the control arm) and extra haematinics according to national guidelines.

## Results

### Enrolment and follow up

**Figure 2:**
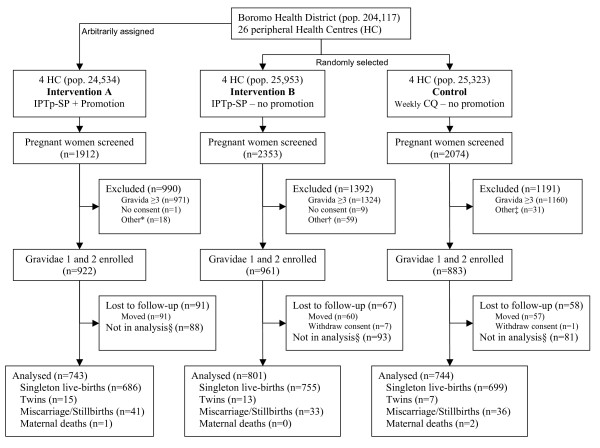
**Study design, enrolment and follow-up of study participants, Boromo Health District, Burkina Faso (2004–2006)**. * 18 near term in March-April 2004. † 3 not pregnant, 1 delivered before enrollment, 55 near term in March-April 2004. ‡ 2 not pregnant, 29 near term in March-April 2004. §delivered before 1^st ^Sept 2004.

Between April 2004 and March 2006, WFA identified 6,339 pregnant women, 2,884 in their first or second pregnancy, 2,766 of whom were enrolled (Figure 2). During the study period, eight women withdrew consent and 208 (8.2%) were lost to follow-up, resulting in 2,550 pregnancies with known outcome. Two hundred and sixty two women delivered before September 1^st^, 2004 and were excluded from the analysis. Six women died, three during pregnancy (one bacterial meningitis despite hospital treatment, one unsafe abortion at home and one unknown disease at home), one at delivery and two after delivery (unknown cause). WFA did 1,780 home visits for blood sampling at around 32 weeks of gestation, covering 77.8% of women with known pregnancy outcomes. Missed visits were mainly due to the high mobility of the study subjects. About two thirds of deliveries took place in a HC (62.7%; 1,434/2,288). At 32 weeks gestation haematological and parasitological data were available for about 70–80% of women, with a similar proportion at delivery for HC deliveries and about 20% for home deliveries. Birth weight within one week after delivery was collected for almost 90% of singleton live-births (Table 1).

**Table 1:** Available outcome measures for primi- and secundigravidae by place of delivery, Boromo Health District, Burkina Faso (2004–2006)

		Place of delivery		
		
Outcome, n (%)	All women	Health Centre	Home	Other
**32 weeks**	n = 2288	n = 1434	n = 806	n = 48*
PCV	1698 (74.2)	1106 (77.1)	583 (72.3)	9 (18.8)
Thick film	1773 (77.5)	1157 (80.7)	607 (75.3)	9 (18.8)
**Delivery**	n = 2285	n = 1434	n = 806	n = 45
PCV any	1696 (74.2)	1100 (76.7)	585 (72.6)	11 (24.4)
PCV < 24 h	1212 (53.0)	1048 (73.1)	158 (19.6)	6 (13.3)
Thick film any	1916 (83.9)	1286 (89.7)	618 (76.7)	12 (26.7)
Thick film < 24 h	1403 (61.4)	1223 (85.3)	173 (21.5)	7 (15.6)
Placental smear	1363 (59.6)	1222 (85.2)	135 (16.7)	6 (13.3)
**Birth weight**†	n = 2140	n = 1364	n = 764	n = 12
< 24 h	1501 (70.1)	1328 (97.4)	166 (21.7)	7 (58.3)
1–8 days	364 (17.0)	32 (2.3)	330 (43.2)	2 (16.7)

PCV = packed cell volume

* including 3 women who died during pregnancy

† only singleton live births

### Women's characteristics

Study participants were mainly young women (mean age 19.7 years) without formal education, mostly married and living within 5 km from the nearest HC. Women in the intervention A arm were slightly older than in the intervention B and control arms and less likely to have a fund raising activity. The wealth index was skewed towards the poorer category in the control arm and towards the less poor in the intervention A arm. Nevertheless, most variables at baseline were comparable between study arms (Table 2).

**Table 2:** Baseline characteristics of study participants (n = 2,288), Boromo Health District, Burkina Faso (2004–2006)

		Study arm		
		
Characteristic	All women n = 2288	Intervention A n = 743	Intervention B n = 801	Control n = 744
Age				
Median (range), years	19 (14–41)	20 (14–41)	19 (15–37)	19 (15–35)
≤ 19 years	54.1	45.9	60.8	55.1
Gravidity				
Primigravidae	55.6	57.1	56.2	53.6
Previous pregnancy outcome if secundigravid †:				
Miscarriage or stillbirth	13.0	15.7	11.7	11.9
Child death*	16.7	12.9	17.4	19.4
Formal education (any school)				
Mother	21.5	25.3	18.1	21.4
Father ‡	26.6	29.1	28.0	22.6
Activity				
Mother with own income	69.3	52.6	79.3	75.3
Father farmer/breeder	92.9	87.9	94.8	93.5
Wealth Index* §				
most poor	22.7	14.2	22.7	31.3
poor	27.2	24.3	24.7	33.0
less poor	24.9	19.6	32.7	22.1
least poor	25.1	42.0	19.9	13.6
Marital status of mother				
Not married	5.1	6.1	4.4	4.8
Religion				
Muslim	48.0	57.5	44.9	41.8
Christian	19.1	22.3	16.5	18.8
Traditional	32.9	20.2	38.6	39.4
Ethnic group*				
Bwaba/Dafing	53.9	12.9	69.2	78.5
Ko/Nounouma	24.1	60.8	10.7	1.9
Mossi	11.3	16.3	11.1	6.6
Fulani	5.5	5.2	7.1	4.0
Other	5.1	4.7	1.9	9.0
Distance from nearest HC (km)				
≤ 5	73.0	76.9	61.3	81.7
> 5	27.0	23.2	28.7	18.3
Bed net*				
Owns bed net at enrolment	30.2	35.1	35.8	19.1

Intervention A = IPTp+promotion; Intervention B = IPTp alone; Control = CQ chemoprophylaxis; HC = health centre

Numbers are proportions if not otherwise stated.

* Proportions significantly different between study arms

† Intervention A n = 319; Intervention B n = 351; Control n = 345

‡ Intervention A n = 690; Intervention B n = 768; Control n = 721

§Intervention A n = 741; Intervention B n = 787; Control n = 737

### Effect of the promotional campaign on IPTp-SP uptake

#### ANC attendance

The great majority (95.6%; 2,187/2,288) of study participants attended ANC at least once during pregnancy, but only half (50.0%; 1,143/2,288) attended three or more times (Table 3). The proportion of women with three or more ANC visits was higher with (intervention A) than without promotion (intervention B and control combined) (OR 2.1 95%CI 0.95–4.65 p = 0.063). About one quarter of the women (26.6%; 569/2,137) had their first ANC visit during the third trimester. This was significantly less frequent in the promotion (18.7%) than in the two non-promotion arms (29.6%) (OR 0.55 95%CI 0.36–0.84 p = 0.011). The proportion of women having received at least 2 doses of SP was significantly higher in the intervention A than in the intervention B arm (69.9% vs. 48.6%; OR 2.45, 95% CI 1.25–4.82 p = 0.014).

**Table 3:** Comparison of main outcome measures by study arm, Boromo Health District, Burkina Faso (2004–2006)

	Study arms			Comparisons		
		
	Intervention					
Characteristic	A	B	Control	A *vs. *Control	B *vs. *Control	A *vs. *B
		
		% (n/N)			OR (95%CI)	
**Antenatal care**						
≥ 3 ANC visits	62.3 (463/743)	42.3 (339/801)	45.8 (341/744)	1.95 (0.86–4.45)	0.87 (0.36–2.10)	2.25 (0.86–5.92)
1^st ^ANC visit in third trimester †	18.7 (127/679)	28.5 (199/699)	30.8 (205/666)	0.52 (0.26–1.03)	0.90 (0.47–1.70)	0.58** (0.44–0.76)
≥ 2 doses IPT-SP	69.9 (519/743)	48.6 (389/801)				2.45* (1.25–4.82)
**Home visit at 32 weeks**						
Anaemia (PCV < 33)	49.4 (288/583)	48.7 (285/585)	56.4 (299/530)	0.75 (0.37–1.54)	0.73 (0.36–1.50)	1.03 (0.68–1.56)
Moderate/severe anaemia (PCV < 30)	23.8 (139/583)	27.7 (162/585)	30.2 (160/530)	0.72 (0.39–1.34)	0.89 (0.45–1.73)	0.82 (0.62–1.08)
Peripheral parasitaemia	20.3 (121/597)	19.4 (120/618)	29.9 (167/558)	0.60* (0.39–0.91)	0.56** (0.40–0.80)	1.05 (0.67–1.67)
**Delivery**						
Anaemia (PCV < 33) ‡	31.1 (137/440)	32.8 (131/399)	40.2 (150/373)	0.67 (0.38–1.18)	0.73 (0.39–1.36)	0.92 (0.51–1.69)
Moderate/severe anaemia (PCV < 30) ‡	16.8 (74/440)	16.8 (67/399)	19.8 (74/373)	0.82 (0.46–1.46)	0.82 (0.46–1.46)	1.00 (0.58–1.72)
Peripheral parasitaemia‡	17.4 (87/499)	20.1 (95/472)	33.3 (144/432)	0.42*** (0.30–0.60)	0.50** (0.37–0.69)	0.84 (0.60–1.18)
Placental parasitaemia	18.1 (89/491)	20.5 (90/440)	30.3 (131/432)	0.51** (0.37–0.70)	0.59* (0.44–0.78)	0.86 (0.58–1.29)
Low birth weight (< 2500 grams) §	16.2 (95/585)	18.6 (128/687)	22.3 (132/593)	0.68 (0.42–1.08)	0.80 (0.46–1.39)	0.85 (0.61–1.17)
Miscarriage/stillbirths ||	5.5 (41/742)	4.1 (33/801)	4.9 (36/742)	1.15 (0.89–1.48)	0.84 (0.55–1.28)	1.36 (0.88–2.12)

Intervention A = IPTp+promotion; Intervention B = IPTp alone; Control = CQ chemoprophylaxis; ANC = antenatal care; PCV = packed cell volume; OR = odds ratio; CI = confidence interval.

The non-adjusted risk estimates are presented as these did not change when adjusted for possible confounders.

* p < 0.05; ** p < 0.01; *** p < 0.001;

† singleton live-births only;

‡ only samples taken the day of delivery;

§ live born singletons weighed within 8 days after delivery;

|| 3 maternal deaths not included

### Effectiveness of IPT-SP with and without promotional campaign

#### Parasitological outcomes

##### Peripheral parasitaemia during pregnancy and at delivery

At 32 weeks of gestation, the prevalence of malaria infections, all *P. falciparum*, was 23.0% (408/1,773) and varied significantly with season, i.e. 35.0% (302/863) during the high transmission period and 11.6% (105/904) during the low transmission period (OR 4.1 95% CI 2.91–5.77 p < 0.001). Primigravidae were more frequently infected than secundigravidae (26.2% vs. 19.5%; OR 1.47 95% CI 1.02–2.11 p = 0.040). Peripheral parasitaemia prevalence at delivery showed a similar pattern with an overall parasite rate of 23.2% (326/1,403), reaching 37.7% (259/688) in the high transmission period compared to 9.0% (64/709) during the low transmission period (OR 6.08 95%CI 3.37–10.98 p < 0.001). Primigravidae were more likely to be infected at delivery than were secundigravidae (28.2% vs. 17.1%; OR 1.90 95%CI 1.41–2.55 p = 0.001).

Malarial infection, both at 32 weeks and at delivery, was significantly more frequent in the control arm (32 weeks 29.9%; delivery 33.3%) than in either of the intervention arms (intervention A: 20.3% and 17.4%; intervention B: 19.4% and 20.1%), although there was no difference between the two IPTp arms. After adjustment for parity and season, the probability of peripheral parasitaemia at delivery was significantly reduced by 68% with intervention A (AOR 0.32 95% CI 0.23–0.44 p < 0.001) and by 61% with intervention B (AOR 0.39 95% CI 0.27–0.57 p < 0.001) as compared to the control arm.

##### Placental parasitaemia

The prevalence of placental malaria was 22.7% (310/1,363), most infections occurring during the high transmission season (38.8% vs.7.1% in low transmission; OR 8.2 95% CI 4.6–14.7 p < 0.001), and in primigravidae (27.6% vs. 16.7% in secundigravidae; OR 1.9 95% CI 1.4–2.5 p < 0.001). The prevalence was lower in the intervention groups (intervention A: 18.1%; intervention B: 20.5%) than in the control group (30.3%). Parity and season adjusted odds ratios (AOR) were 0.38 (95% CI 0.28–0.51 p < 0.001) for intervention A and 0.47 (95% CI 0.32–0.70 p = 0.002) for intervention B compared with the control group. The difference between intervention A and intervention B was not significant (AOR 0.79 95%CI 0.51–1.23 p = 0.268).

#### Haematological outcomes

##### Anaemia during pregnancy and at delivery

**Figure 3:**
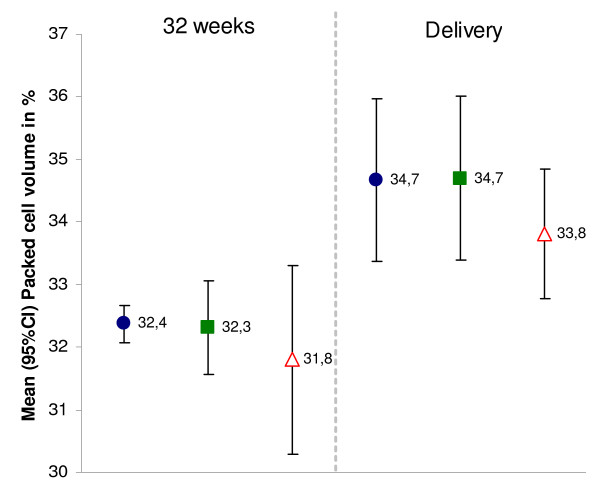
**Mean PCV during pregnancy and at delivery by study arm, Boromo Health District, Burkina Faso (2004–2006)**. Intervention A = blue ●; Intervention B = green ■; Control = red ∆. Numbers next to symbols represent point estimates of the mean; error bars represent 95% confidence intervals.

Overall mean PCV was 32.2 (95%CI 31.6–32.8) at 32 weeks gestation and 34.4 (95%CI 33.6–35.2) at delivery and was lower, though not significantly, in the control arm compared with the two intervention arms combined (Figure 3).

Risk factors for anaemia (PCV < 33) at 32 weeks in univariate analysis were primiparity (OR 1.33 95%CI 1.06–1.66 p = 0.016) and high transmission season (OR 1.57 95%CI 1.19–2.09 p = 0.005), bed net ownership had a protective effect (OR 0.81 95%CI 0.7–0.93 p = 0.007). At delivery, risk factors for anaemia included primiparity (OR 1.41 95%CI 1.07–1.84 p = 0.018), season (OR 1.5 95%CI 1.08–2.07 p = 0.019) and poor wealth index (OR 1.42 95%CI 1.09–1.85 p = 0.014).

The prevalence of anaemia (PCV < 33) was higher in the control arm (32 weeks: 56.4%; delivery: 40.3%) than in the intervention arms (intervention A: 49.4% and 30.8%; intervention B: 48.7% and 32.7%), but none of the differences in pair-wise and grouped (intervention A and B vs. control) comparisons reached statistical significance (Table 3). Differences between study arms remained non significant after adjustment for parity, season, bed net ownership and wealth index.

The prevalence of severe anaemia (PCV < 24) was below 5%, both at 32 weeks (2.9%, 95%CI 1.9–3.9) and at delivery (3.2%, 95%CI 1.9–4.9) and did not significantly differ between study arms.

#### Birth outcomes

**Figure 4:**
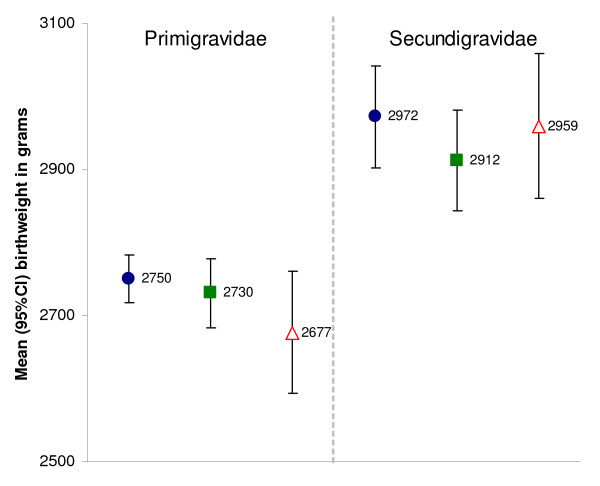
**Mean birth weight of live-born singletons by study arm, Boromo Health District, Burkina Faso (2004–2006)**. Intervention A = blue ●; Intervention B = green ■; Control = red ∆. Numbers next to symbols represent point estimates of the mean; error bars represent 95% confidence intervals.

Overall mean birth weight of live singletons was 2,822 g (95%CI 2,782–2,862); 2,720 g (95%CI 2,674–2,766) for primigravidae and 2,945 g (95%CI 2,893–2,997) for secundigravidae (p < 0.001). LBW was associated with placental malaria only in primigravidae (OR 2.4 95%CI 1.5–3.9 p = 0.002) but not in secundigravidae (OR 0.84 95%CI 0.37–1.93 p = 0.661). Mean birth weight did not significantly differ between study arms (Figure 4) though the prevalence of LBW compared to weekly CQ was reduced by 27% (from 22.3 to 18.6) with intervention A and by 17% (from 22.3 to 16.2) with intervention B (Table 3). However, even after adjusting for parity, season, sex of the baby, distance, and bed net ownership none of these differences was significant (intervention A vs. control: AOR 0.64 95%CI 0.38–1.10 p = 0.097; intervention B vs. control: AOR 0.71 95%CI 0.37–1.35 p = 0.271).

The proportion of miscarriage or stillbirth among women with known pregnancy outcome was not significantly different between study arms.

## Discussion

In this community-based trial, the effect of providing IPTp-SP through routine ANC coupled with promotional activities targeted to pregnant women was assessed by comparing parasitological, haematological, and birth weight outcomes in primi- and secundigravidae. At the time this study was implemented, the national prevention policy for pregnant women was weekly CQ chemoprophylaxis and the Burkinabe Ministry of Health had requested more local data on IPTp-SP prior to changing the policy. Therefore, the effectiveness of IPTp-SP was measured by comparing two intervention arms where HC provided IPTp-SP with a control arm where HC offered weekly CQ while the added value of the promotional campaign targeted to pregnant women was assessed between intervention arms with and without promotion. Despite good coverage with more than two thirds of primi- and secundigravidae in the intervention villages with promotion having taken at least two SP doses, no significant improvement in the haematological and birth weight outcomes was observed. It should be noted that when the effect of the IPTp-SP is analysed at individual level (and not by community), women having received two or more SP doses had a significantly lower risk of peripheral and placental parasitaemia (10.8% and 12.2% respectively), anaemia (13.7% PCV < 30%) and low birth weight (12.8%). This indicates that a major impact of IPTp-SP at community level can be detected only when, as with immunization programmes, the coverage is extremely high. In this setting, reaching the current RBM goal of 80% by 2010 [11] is unlikely to result in a major impact. The critical threshold of coverage yet needs to be defined, possibly on a regional basis. These results also illustrate the difficulty of translating an efficacious intervention into an effective policy.

IPTp-SP performed better than weekly CQ in clearing peripheral and placental parasitaemia, even at a relatively low coverage (2 or more SP doses < 50% without promotion). This finding strongly supports the policy change to IPTp-SP in Burkina Faso, decided in early 2005 [30]. The remaining relatively high rates of placental malaria occurring mainly during the high transmission season in the IPTp-SP groups are consistent with other studies in the region and are likely to be due to high rates of reinfection after the last dose of SP [14,15,31] rather than to SP-resistance. Resistance to SP in urban and rural settings was not widespread at the time the study was conducted [23,24] and it is unlikely that local differences in SP resistance in the study area have significantly influenced the results.

Maternal anaemia and LBW, two major consequences of malaria infection during pregnancy, were only marginally affected by the intervention. Evidence that IPTp-SP reduces anaemia and increases birth weight in primi- and secundigravidae is mainly based on trials where the control group received no treatment [8]. Only three studies directly compared IPTp-SP with weekly CQ, all in women of low parity. In Malawi, a 70% decrease in placental malaria with a mean gain in birth weight of 116 grams was observed, with no data on anaemia reported [13]. In Mali, a moderate reduction of placental malaria with a mean birth weight gain of 33 grams and 30% reduction in anaemia was reported, but with no effect on severe anaemia [14]. Recently, in Burkina Faso, in an urban setting where placental malaria prevalence was below 6%, no difference in mean haemoglobin and birth weight between IPTp-SP and weekly CQ was found [32]. Overall, the greatest impact of IPTp-SP on malaria prevalence has been observed in East Africa, where CQ resistance was high. The smaller difference between IPTp-SP and weekly CQ observed in West Africa, for example in Mali or Burkina Faso, suggests that weekly CQ chemoprophylaxis may still have some preventive effect, reducing then the difference between intervention and control groups. Indeed, in the Burkina Faso capital, Ouagadougou, adequate clinical and parasitological response at day 28 post-treatment in pregnant women treated with CQ was above 50% [23]; the success rate in rural communities, such as those involved in the current study, is probably higher [33]. Therefore, the remaining effectiveness of CQ chemoprophylaxis in the control group may explain the little effect on anaemia and birth weight observed in both intervention groups. This is consistent with the strong association between non-attendance at ANC and peripheral parasitaemia at any time during pregnancy observed in cross-sectional surveys undertaken before the introduction of IPTp in the same study area and with recent data from neighbouring Benin [34,35].

Antenatal care providers in HC where IPTp-SP was implemented were advised to give two doses of SP to pregnant women at the beginning of the second and third trimester as recommended by the WHO guidelines [36]. Since the start of the study, recommendations have been modified and simplified; "beginning of the second trimester" has been replaced by "after quickening", and "third trimester" by "at least one month apart". This change leaves uncovered the period between the beginning of the second trimester and the onset of quickening. Most countries implementing IPTp have chosen the two-dose-policy because the evidence for a beneficial effect of a third dose was limited to HIV-positive women [37]. In the current WHO recommended schedule of four ANC, with three visits after quickening, the frequency of IPTp dosing is no longer limited to two doses (and three where HIV-prevalence is >10%), but IPTp should be given "with each scheduled visit after quickening" to "assure that a high proportion of women receive at least two doses" [1]. These simplified recommendations take into account the difficulty to assess gestational age in busy peripheral ANC and to decide whether to administer SP. In the present study, giving SP at every ANC visit after the first trimester would have resulted in a better coverage with 90% women with more than two doses (instead of 70%), and 61% with more than three doses (instead of 4%) in the intervention A arm.

This is the first community-based effectiveness trial on IPTp-SP examining ways of increasing SP uptake through existing health services. It offers an interesting insight on the difficulties of increasing IPTp coverage. Alternative delivery systems of IPTp within the community have recently been tested in Uganda and Malawi. In both studies women receiving SP from community sources less frequently completed ANC, a side effect that may have a negative impact on women's health (K. Msyamboza, personal communication) [38]. In the present study, participants were enrolled at community level but the randomization unit was the health centre as the main intervention (IPTp-SP) was related to the ANC. Therefore, variations between health centres in number of communities and population covered, as well as mobility and experience of the health staff, may have contributed to a larger cluster design effect than initially expected, a limitation of the study. For the intervention, the selection of health centres was not done randomly but on geographical contiguousness to avoid contamination (promotion campaign) between study arms. While known differences in baseline characteristics (age, ethnic group, wealth index) could be adjusted for, unknown factors may have influenced the findings although it is unlikely this would have resulted in an important bias. Moreover, the difficulties of following up pregnant women in a highly mobile rural population, often with sub-optimal working conditions in health centres and with a substantial number of home deliveries, should be appreciated. Despite substantial logistic constraints, our data include both health centre and home deliveries, and are more likely to reflect "real life conditions" than most health centre based studies.

It is noteworthy that this study found similar prevalences for peripheral and placental parasitaemia while the actual prevalence of parasitaemia is generally underestimated by peripheral microscopy [39]. The staff in peripheral health centres was trained to remove excess blood when preparing placental smears and excessively dried samples may have limited the number of red blood cells on the slide thus reducing the sensitivity of placental microscopy. Moreover, it was not possible to blind microscopists to the peripheral blood results, a further limitation.

## Conclusion

The promotional campaign resulted in a major increase in coverage, with two thirds of women receiving at least two SP treatments during pregnancy. Despite their lower prevalence of malaria infection this did not translate into a difference in maternal anaemia or birth weight outcomes because the remaining proportion (30%) of primi- and secundigravidae not having received two SP treatments dilute the impact of the intervention. SP administration at all ANC visits following the first trimester should dramatically increase coverage. Since differences between groups with 49% and 70% SP coverage were only marginal, it is unclear what impact could be observed if the current RBM goal of 80% by the year 2010 is reached. This data provides evidence that, as with immunization programmes, extremely high coverage is essential for effectiveness. This critical threshold of coverage needs to be defined, possibly on a regional basis. On-going active surveillance of SP uptake should be an important component of all malaria in pregnancy control activities.

## Competing interests

The authors declare that they have no competing interests.

## Authors' contributions

UDA, BJB and SOC designed the study. SG and SOC were responsible for the implementation of the study and data collection with help from FTO. CK was responsible for the design and supervision of the promotional activities with help from SG and SOC. SG did the data management and statistical analyses. SG and UDA wrote the manuscript with major contributions of the other authors. All authors read and approved the final manuscript.
